# Metal‐Induced Tuning of Fullerene Reactivity: Application to Nucleophile Addition

**DOI:** 10.1002/cphc.202500487

**Published:** 2025-11-03

**Authors:** Corentin Rossi, Anne P. Rasmussen, Bérenger Gans, Ugo Jacovella

**Affiliations:** ^1^ Institut des Sciences Moléculaires d’Orsay Université Paris‐Saclay, CNRS 91405 Orsay France; ^2^ Department of Physics and Astronomy Aarhus University Ny Munkegade 120 8000 Aarhus C Denmark

**Keywords:** fullerenes, gas‐phase reactions, isomers, kinetics, mass spectrometry

## Abstract

Endohedral metallofullerenes (EMFs) are a unique class of hybrid molecules formed by encapsulating metal atoms within carbon cages (fullerenes), giving rise to distinctive properties that differ from empty fullerenes. Extensive research has focused on optimizing the synthesis, extraction, isolation, and characterization of EMFs, along with investigating their physicochemical properties and potential applications in areas such as electronics, photovoltaics, biomedicine, and materials science. Here, the use of a laser vaporization source combined with ion mobility and mass spectrometry is demonstrated to characterize and isolate EMF structures, enabling further investigation of their gas‐phase chemical properties. This approach is illustrated through a comparative study of the reactivity of empty carbon cages and calcium EMFs in the nucleophilic addition of pyridine.

## Graphical abstract

A textbook case of how encapsulating an atom in a carbon cage can tune assembly reactivity based on partial charge distribution–exemplified by the classic nucleophilic attack.

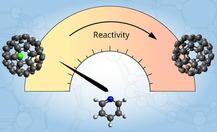

## 1 Introduction

Endohedral metallofullerenes (EMFs) have established themselves as fascinating and unique species. They have been subject to many review articles during the past 25 years (see^[^ ^1^, ^2^, ^3^, ^4^, ^5^, ^6^, ^7^, ^8^, ^9^ ^]^ and refs. therein). The encapsulation of metal atoms inside fullerene cages imparts distinct chemical properties, differing significantly from both empty fullerenes and free metal atoms. This encapsulation results in altered reactivity and unique electronic characteristics, which are typically studied in the condensed phase (e.g., ref. [10]) and remain a challenge to investigate in the gas phase. The fullerene cage also protects the encapsulated metal atom from the external environment, preventing oxidation or other typical chemical reactions and thereby enhancing its stability. The interaction between the metal atoms and the carbon cage grants EMFs with unique electronic and magnetic properties, which can be tuned by varying the type of encapsulated metal atom. Consequently, EMFs have been explored in diverse research fields, including electronics (tested as transistors, sensors, and other nanoelectronic devices), photovoltaics (solar cells and other photovoltaic devices), biomedicine (as drug delivery agents or magnetic resonant imaging (MRI) contrast agents), and materials science (designing new materials with specific electronic, magnetic, or optical properties).^[^ ^1^, ^2^, ^3^, ^4^, ^5^, ^6^, ^7^, ^8^, ^9^ ^]^ Interest in EMFs also extends to the astrophysics community, as they are hypothesized to exist in space and potentially play a significant role in interstellar chemical evolution.^[^ ^11^, ^12^, ^13^, ^14^, ^15^ ^]^

Typically, EMFs are synthesized in the gas phase from high‐temperature, high‐density carbon vapor by evaporating metal‐doped graphite. All production methods involve generating a carbon plasma with co‐evaporated metal in the presence of a buffer gas. The most prevalent and widely used method for producing EMFs is the arc‐discharge Krätschmer–Huffman reactor, known for achieving the highest yield.^[^ ^1^, ^2^, ^3^, ^4^, ^5^, ^16^, ^17^ ^]^ This method involves passing a high current through graphite material containing a small quantity of the desired metal. The result is a soot, which is subsequently extracted using either solvent or electrochemical methods, followed by separation with high‐performance liquid chromatography (HPLC). However, extraction of EMFs is challenging because the EMFs produced in soot are typically nonsoluble in common fullerene solvents. While other solvents can be used for extraction, they are not suitable for HPLC purification. The hardship in extraction and purification has, so far, impeded comprehensive structural and electronic studies of EMFs. When EMFs are intended for investigation via mass spectrometry or laser spectroscopy, they are typically generated using a laser vaporization source. In this process, a pulsed laser irradiates a moving graphite target containing a small amount of metal, which also allows the study of growth mechanisms, as demonstrated by Kroto and coworkers.^[^ ^18^ ^]^

The typical methods for elucidating the structure of metallofullerenes in the gas phase, whether the metal resides inside the cage or is externally bound to it, are through ion‐molecule reactions,^[^ ^19^ ^]^ dissociation using lasers,^[^ ^20^, ^21^ ^]^ or collisions.^[^ ^18^, ^22^, ^23^ ^]^ Yet, the ultimate tool is ion‐mobility spectrometry, which provides direct access to information related to the shape of the species of interest. To our knowledge, ion‐mobility spectrometry has only been utilized for the cases of lanthanum, niobium, and scandium‐doped fullerenes by the group of Jarrold^[^ ^24^, ^25^, ^26^, ^27^, ^28^, ^29^ ^]^ and for anionic large EMFs by the group of Kappes.^[^ ^30^ ^]^

In this article, we synthesize gas‐phase EMFs containing calcium (Ca) atoms using a laser vaporization technique (see Methods and ref. [31]). In previous studies, cage structures were inferred based on the absence of odd‐carbon clusters hosting a metal (CaC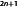), while the endohedral nature of Ca@C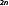 species (@ for endohedral) was attributed to the observation that these clusters fragment by losing a C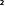 unit rather than the metal atom.^[^ ^18^, ^32^, ^33^, ^34^ ^]^ After confirming the structure of Ca–EMFs using ion‐mobility spectrometry and isolating them, we demonstrate how the encapsulated metal influences the chemical properties of the fullerene cage through a prototypical nucleophilic addition reaction with pyridine.

## 2 Results and Discussion

First, we will demonstrate how to elucidate the structure of metallocarbon species, enabling the identification of EMFs. Next, we will show how a specific EMF can be isolated to study its reactivity and compare it to that of the corresponding empty fullerenes, allowing us to assess the chemical influence of the encapsulated metal atom.

### 2.1 Structural Analysis

**Figure 1:**
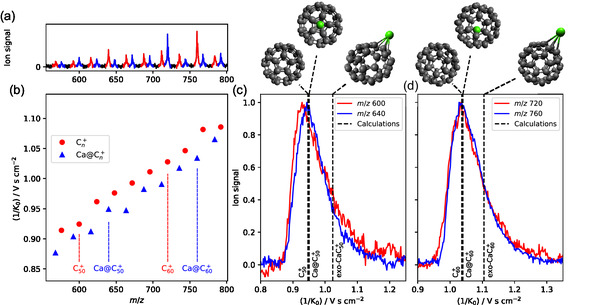
Structural analysis of Ca‐containing cages. a) Mass spectrum of Ca‐doped graphite, with red and blue peaks highlighting bare carbon and Ca carbide species, respectively. b) Corresponding ion‐mobility spectrum, plotted as inverse reduced mobility versus mass‐to‐charge ratio. Only the maximum of mass‐selected ion distributions is reported. c,d) ATD of m/z (c) 600, 640, (d) 720, and 760, along with CCS calculations (dashed line) for C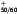, Ca@C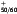, and exo‐CaC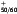. Computational details for CCS determination are given in the Supplementary Information.

Panel (a) in **Figure** **1** presents the mass spectrum, with bare carbon clusters highlighted in red ($m/z({C_{2n}}^{+})$) and Ca‐containing carbon clusters in blue ($m/z{\left(C_{2n}\right)}^{+}+40$). While the mass spectrum alone does not provide structural information, panel b) in Figure 1 shows the mobility spectrum, enabling further ion discrimination based on shape.

Ion‐mobility spectrometry separates ions according to their collision cross sections (CCS) with a buffer gas (N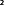 in this work); compact ions travel faster through the gas, reaching the detector earlier than more extended ones. The similar arrival times of C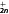 and CaC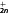 species suggest that calcium is most likely encapsulated within the cage, forming an EMF, rather than attaching externally.

Figure 1c provides the arrival time distribution (ATD) of the ions at m/z 600 and 640, corresponding to C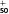 and CaC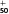, where the *y*‐axis corresponds to the ion signal, and the *x*‐axis corresponds to inverse reduced mobilities (proportional to the CCS). Panel (d) displays the same data for ions at m/z 720 and 760, corresponding to C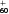 and CaC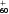. The calculated inverse reduced mobility values (dashed lines) for the bare fullerenes, the exohedral metallofullerenes, and the EMFs further confirm the formation of EMFs containing Ca, with no evidence of exohedral structures.

An extended version of Figure 1 panel (b) is shown in the Supplementary Information, covering the m/z 600–1400 range. The size distribution of Ca‐containing species shifts noticeably toward smaller cages compared to bare carbon clusters, with CaC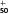 becoming nearly as abundant as CaC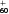. In our instrument, the smallest cage capable of accommodating a Ca atom was found to be C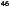.

### 2.2 Chemical Properties Analysis

**Figure 2:**
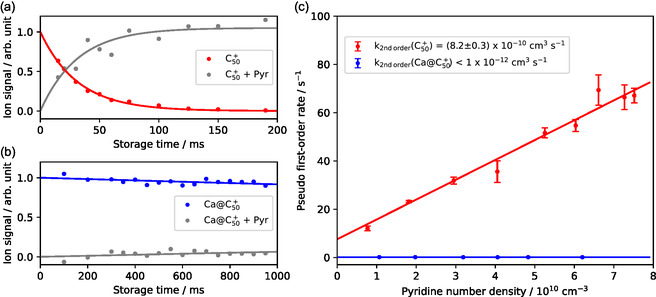
Typical kinetic profiles for reactions C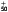 and Ca@C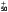 with pyridine are displayed in panels a) and b), respectively. c) Pseudo first‐order reaction coefficient versus pyridine number density, used to extract second‐order rate coefficients, is presented in panel. Error bars represent the statistical error from the exponential fit of the kinetic profile.

Isomer‐selected ion‐molecule ([M]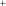) reactivity was investigated at 300 K in an octupole‐ion trap, focusing on the nucleophilic addition of pyridine to carbon cages. The selected species react with pyridine to form [M + 79]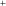 adducts, where pyridine is introduced in large excess compared to the number of ions. Differences in the reactivity of C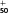 and Ca@C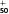 with pyridine are illustrated in **Figure** **2**.

At a given concentration of pyridine, the ion signals of the reactants and products were recorded as a function of reaction time (storage time), leading to kinetic curves as presented in panels (a) and (b). These kinetic curves were obtained at various pyridine number densities, giving pseudo‐first‐order rates from the mono‐exponential fit of the decay of the parent ions. The second‐order rate coefficient is then retrieved by plotting the pseudo‐first‐order rate coefficient against pyridine density within the ion trap. The slope obtained from the linear regression indicates the second‐order rate coefficient, as shown in Figure 2c. This illustrates that the second‐order rate coefficient is at least two orders of magnitude slower when the Ca atom is inside the C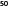 cage. We then investigated whether a trend could be identified in the reactivity quenching caused by encapsulating the Ca atom within the cages.

**Figure 3:**
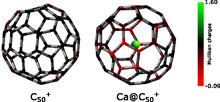
Mulliken charges calculated for C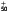 (left) and Ca@C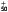 (right) using density functional theory (B3LYP/6‐31G(d)).

The general low reaction rates of EMFs prevented the systematic extraction of second‐order rate coefficients. Thus, we decided to record only the mass spectra obtained with a specific trapping time of 75 ms at a given pyridine number density ($10^{10}$ cm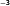), as shown in the Supporting Information. This demonstrated a notable reduction in reactivity, ranging from 70% to 90% depending on the cage size, upon the addition of a Ca atom. Our mobility resolution does not allow us to distinguish small differences in the cage structure that could account for minor changes in reactivity on the order of a few percent. Similarly, encapsulation of the cage can slightly perturb its structure compared to the corresponding empty cages. Nevertheless, the dominant quenching effect arises from charge transfer between the metal and the cage. This can be understood through basic principles of organic chemistry. For example, C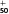 is a well‐known electrophile and readily undergoes nucleophilic addition, particularly with pyridine, which has a high proton affinity. In contrast, for Ca@C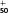, the encapsulated Ca atom is expected to adopt a +II oxidation state. A simple Mulliken charge analysis shows that Ca carries a partial charge close to +1.5, inducing the C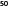 cage to take on a negative character to maintain the overall +1 charge, as illustrated in **Figure** **3**. This species is significantly less electrophilic than C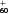, explaining the marked decrease in reactivity toward pyridine.

## 3 Conclusion

This study experimentally highlights the distinct gas‐phase chemical properties of EMFs in comparison to their empty fullerene counterparts. Encapsulation of Ca atoms within fullerene cages leads to significant reductions in reactivity, as evidenced by nucleophilic addition reactions with pyridine. These findings demonstrate how encapsulation alters the electronic environment of the carbon cage, specifically reducing its electrophilicity. The encapsulation of a metal could be used to shield the cage from surrounding nucleophilic species. The results advance our understanding of how metal encapsulation can tailor fullerene properties, providing valuable insights for applications in diverse fields. Moreover, this work demonstrates the ability of our instrument to investigate EMFs as versatile platforms for exploring novel chemical phenomena and potential applications.

## Supporting Information

The authors have added additional data to support the findings of this study and have cited additional references in the supporting information.^[31,35–39]^

## Conflict of Interest

The authors declare no conflict of interest.

## Supporting information

Supplementary Material

## Data Availability

Raw and processed supporting the findings of this article are openly available on Zenodo at https://doi.org/10.5281/zenodo.17038849.
