# Supplementary material for: Metal‐Induced Tuning of Fullerene Reactivity: Application to Nucleophile Addition
Source: Chemphyschem. 2025 Nov 3;26(23):e202500487. doi: 10.1002/cphc.202500487 (PMC12677715; doi:10.1002/cphc.202500487)
Supplement: Supplementary file 1 — Supplementary Material [file CPHC-26-e202500487-s001.pdf]

Metal-Induced Tuning of Fullerene Reactivity: Application to Nucleophile Addition

Corentin Rossi, Anne P. Rasmussen, Bérenger Gans, Ugo Jacovella*

Methods

This apparatus and its performance to study isomer–selected ion–molecule reactivity is detailed in Ref. 1. Only the main details are outlined here. The setup is divided into four main parts: the ion source, the drift region (for shape selection), the reaction chamber, and the quadrupole mass filter (for mass selection). The metal carbide ions are generated by laser vaporization of a rotating carbon rod soaked in a saturated metallic $CaCl_2$ salt water solution using the focused, frequency-doubled output of a nanosecond pulsed Nd:YAG laser (532 nm) at 20 Hz repetition rate for recording the mobility spectra and 1 and 5 Hz for the reactivity experiments of $C_{50}Ca$ and C_{50}, respectively. Metal carbide ions are then propelled by an electric field in a drift tube made of highly resistive glass through a nitrogen buffer gas ($\approx$ 2 mbar), causing them to be separated spatially and temporally based on their collision cross sections with the buffer gas. More extended ions exohedral fullerenes have larger collision cross sections and move through the drift tube more slowly, while more compact ions such as EMFs, which collide less, travel faster. The ions are then collected by a radio-frequency (RF) ion funnel at the exit of the drift tube and passed through a small orifice (0.8 mm) of a skimmer-shape electrode into an octupole RF ion guide (pressure of $\approx 1 \times 10^{-4}$ mbar). The ions travel from the octupole through a 3-mm aperture in a set of ring electrodes into the quadrupole mass filter (pressure of $\approx 1 \times 10^{-6}$ mbar), where they undergo mass selection. Following mass-selection in the quadrupole mass filter, the ions can be deflected to an off-axis ion detector to obtained mobility spectra.

Structural analysis

Ion-mobility spectra are obtained by measuring the arrival-time distribution (ATD) as a function of the mass-to-charge ratio, which is filtered by the quadrupole, as presented in Fig. S1. The arrival times are then converted to reduced mobility to enable comparison with other experiments using the following equations:

$$K = \frac{1}{t} \cdot \sum_i \frac{l_i^2}{V_i} \tag{1}$$

$$K_0 = K \cdot \frac{P}{1013.25} \cdot \frac{273.16}{T} \tag{2}$$

where t refers to the time ions spend in the drift region, l_i and V_i refer to the length and the voltage drop of the different sections of the experimental setup, T and P the experimental temperature and pressure in the drift region in kelvin and millibar, respectively.[2,3]

Measured mobilities are calibrated with respect to the reported experimental value for C_{60}^+ (213.1 Å^2, Ref. 4). To analyze the structural features responsible for the ion signals observed in the ATDs, we can calculate their theoretical reduced mobility by determining their collisional cross section with N_2. This process involves three steps: (i) geometry optimization, (ii) calculation of partial charges (in this paper using Merz-Kollman theory), and (iii) calculation of the collision cross section. Steps (i) and (ii) were performed using the Gaussian 16 software package[5] at B3LYP/6-31G level of theory, while step (ii) was done using the Ion Mobility Software (IMoS) 2.0 program suite[6] with the trajectory method parametrized for N_2 buffer gas at 300 K.

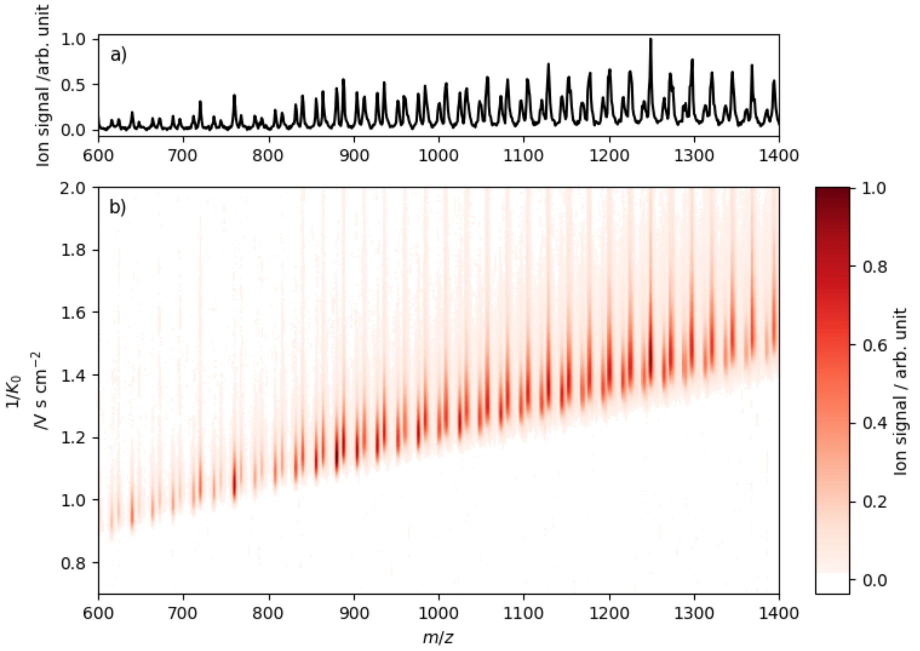

Figure S1: Mass spectrum of Ca-doped graphite. (b) Corresponding ion mobility spectrum, plotted as inverse reduced mobility *versus* mass-to-charge ratio.

Chemical properties analysis

The skimmer is polarized and can serve as an ion gate, which can be precisely controlled to open with a specific delay and duration. This allows for the selective transmission of desired isomers, while blocking those that are slower or faster. Isomer-selected ion-molecule reactions can be performed in the octupole transfer guide, which can be converted into an ion trap by applying a blocking potential to the exit electrode. The neutral reactant is continuously introduced through a needle valve, with pressure monitored using a Baratron (CMR 375, Pfeiffer Vacuum). The reaction products can then be mass-analyzed using a quadrupole mass filter. The ablation laser was operated at 1 Hz repetition rate giving a dynamical trapping time range of 10 to 995 ms.

Reactivity with other EMFs

We present in Fig. S2 mass spectra obtained with a specific trapping time of 75 ms at a given pyridine density (10^{10} molecules·cm^{-3}). In the left column of Fig. S2, we present the mass spectra of isomeric species selected based on their mobilities. To maintain a sufficiently high ion count, the selection gate, when positioned to select a specific species, may also include a small number of nearby drifting molecules. Additionally, for species such as C_{50}^+ and Ca@C_{50}^+, which have similar mobilities, both are inevitably present in the trap. The left column also demonstrates that, when no pyridine is introduced into the octupole chamber, no mass corresponding to the adducts appear in the mass spectra.

The right column presents mass spectra recorded with pyridine introduced. Red peaks represent the bare carbon cages and their corresponding adducts, while blue peaks indicate Ca-encapsulated fullerenes and their respective adducts.

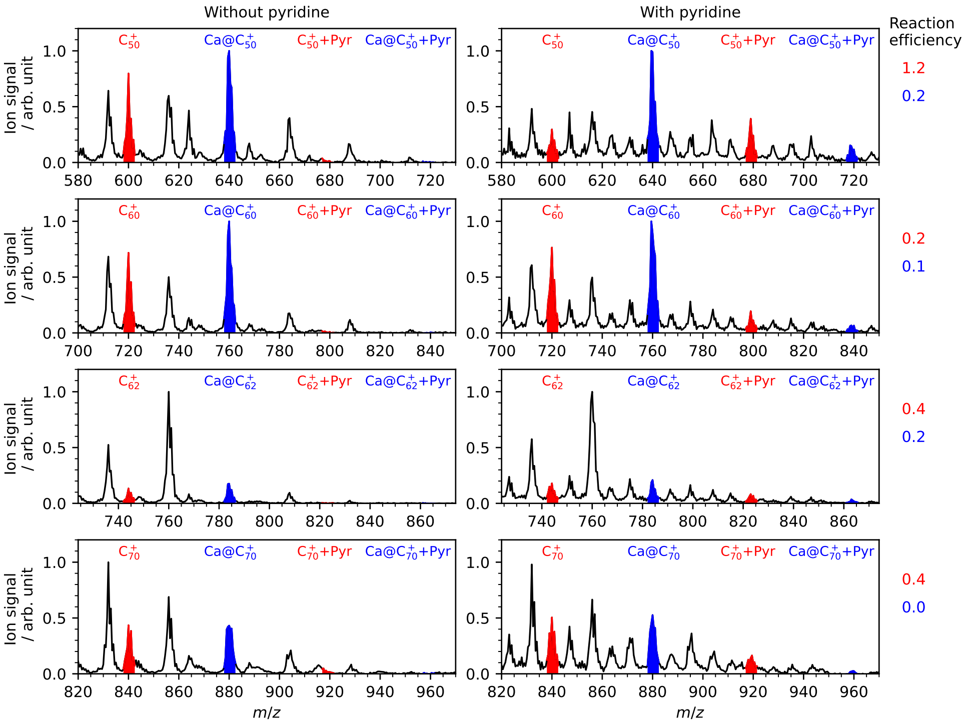

Figure S2: The left column displays representative mass spectra of ionic species selected at the drift time corresponding to C_{50}^{+}/Ca@C_{50}^{+}, C_{60}^{+}/Ca@C_{60}^{+}, C_{62}^{+}/Ca@C_{62}^{+}, and C_{70}^{+}/Ca@C_{70}^{+}. The right column presents mass spectra obtained at a reaction time of 75 ms and a pyridine concentration of 10^{10} molecules·cm^{-3}. Each mass spectrum was normalized to 1 of its maximum. Red peaks represent bare carbon cages and their corresponding reaction adducts, while blue peaks indicate Ca EMFs and their associated adducts.

References

[1] C. Rossi, A. P. Rasmussen, B. Gans, J. Jašík, J. Žabka, M. Albaret, H. Bauduin, C. Charrière, J.-P. Dugal, J. Guigand, C. Le Bris, U. Jacovella, *Chem. Methods* **2025**, *5*, e202500013.

[2] J. Moseley, I. Gatland, D. Martin, E. McDaniel, *Phys. Rev.* **1969**, *178*, 234.

[3] P. Dugourd, R. Hudgins, D. Clemmer, M. Jarrold, *Rev. Sci. Instrum.* **1997**, *68*, 1122.

[4] I. Campuzano, M. F. Bush, C. V. Robinson, C. Beaumont, K. Richardson, H. Kim, H. I. Kim, *Anal. Chem.* **2012**, *84*, 1026.

[5] M. J. Frisch, G. W. Trucks, H. B. Schlegel, G. E. Scuseria, M. A. Robb, J. R. Cheeseman, G. Scalmani, V. Barone, G. A. Petersson, H. Nakatsuji, X. Li, M. Caricato, A. V. Marenich, J. Bloino, B. G. Janesko, R. Gomperts, B. Mennucci, H. P. Hratchian, J. V. Ortiz, A. F. Izmaylov, J. L. Sonnenberg, D. Williams-Young, F. Ding, F. Lipparini, F. Egidi, J. Goings, B. Peng, A. Petrone, T. Henderson, D. Ranasinghe, V. G. Zakrzewski, J. Gao, N. Rega, G. Zheng, W. Liang, M. Hada, M. Ehara, K. Toyota, R. Fukuda, J. Hasegawa, M. Ishida, T. Nakajima, Y. Honda, O. Kitao, H. Nakai, T. Vreven, K. Throssell, J. A. Montgomery, Jr., J. E. Peralta, F. Ogliaro, M. J. Bearpark, J. J. Heyd, E. N. Brothers, K. N. Kudin, V. N. Staroverov, T. A. Keith, R. Kobayashi, J. Normand, K. Raghavachari, A. P. Rendell, J. C. Burant, S. S. Iyengar, J. Tomasi, M. Cossi, J. M. Millam, M. Klene, C. Adamo, R. Cammi, J. W. Ochterski, R. L. Martin, K. Morokuma, O. Farkas, J. B. Foresman, D. J. Fox, Gaussian 16 Revision C.01 **2016**, gaussian Inc. Wallingford CT.

[6] V. Shrivastav, M. Nahin, C. J. Hogan, C. Larriba-Andaluz, *J. Am. Soc. Mass Spectrom.* **2017**, *28*, 1540.
